# A Case Study in Process Improvement to Minimize Delays from Obtaining Blood for Red Cell Exchange for a Patient with Sickle Cell Disease and Multiple Red Blood Cell Alloantibodies

**DOI:** 10.1155/2022/1562921

**Published:** 2022-01-11

**Authors:** Damodaran Narayanan, Noreen B. Hogan, Karen A. Schaser, Patricia Ruegsegger, William Nicholas Rose

**Affiliations:** ^1^Department of Pathology, University of Wisconsin Hospital, 600 Highland Ave Madison, WI 53792, Madison, USA; ^2^Department of Nursing, University of Wisconsin Hospital, 600 Highland Ave Madison, WI 53792, Madison, USA

## Abstract

The process of procuring several units of red blood cells for red cell exchange can sometimes take several hours to days, especially for patients with multiple clinically significant red cell alloantibodies. This can introduce delays, inconveniences, and even health challenges for the patient. For most planned exchanges, these delays are preventable with some foresight and process modifications that are relatively minor yet high leverage. We report a case study of process improvement whereby the apheresis nurse sends an e-mail to the blood bank when the nurse makes the patient's next red cell exchange appointment as the signal to order blood about 6–8 weeks before the exchange.

## 1. Introduction 

Sickle cell disease (SCD) is a common inherited red blood cell (RBC) disorder in the United States, as it affects around 100,000 people and approximately one in 500 African Americans [1, 2]. A common treatment is red cell exchange (RCE), in which about 70% of the patient's blood is replaced by donor RBCs with normal hemoglobin A [1, 2]. One key advantage of RCE over simple transfusion is the prevention of iron overload [1].

In SCD patients, identifying compatible blood products for RCE is important for several reasons. One, SCD patients tend to develop alloantibodies to RBC antigens more frequently (∼30%) than do non-SCD patients who are transfused (2–5%) [3, 4].

Two alloimmunized SCD patients who have a delayed hemolytic transfusion reaction (DHTR) may develop the rare but potentially fatal hyperhemolysis syndrome (HHS). HHS includes hemolysis of both transfused and native RBCs for poorly understood reasons [5]. Some cases of HHS also happen independent of alloimmunization [6].

Three, in order to prevent alloantibodies/DHTRs (and, more importantly, the rare complication of HHS that is sometimes associated with a DHTR), extended phenotype matching is often performed [7–9].

In sum, a significant amount of time is often required to obtain RBCs for SCD patients in advance of RCE [10].

## 2. Case Report

We introduce the patient that was the impetus for our process change. To be clear, we do not claim that the details of the patient alone warrant a case report. Rather, we emphasize that the main purpose of this article is to share the process improvement that was implemented to maximize the quality of care for this patient.

Our patient is a 52-year-old female with hemoglobin SS disease who receives red cell exchanges approximately every 6–8 weeks. She has 5 red cell alloantibodies to the C, E, Fy^a^, Fy^3^, and Js^a^ antigens. The anti-Fy^3^ is particularly challenging, as essentially 100% of the white population has this antigen.

She also has a warm autoantibody that adds extra time for antibody testing and crossmatching. Finally, she also receives K-negative RBCs to prevent alloimmunization, as this practice of extended phenotype matching is common for patients with SCD. Thus, the patient requires RBC units for RCE that are negative for 6 antigens: C, E, K, Fy^a^, Fy^3^, and Js^a^. About 1% of blood donors in the US are negative for all 6 antigens [11].

Her hematologist informed us that she had multiple experiences with the following type of delay in procuring blood. She would visit our apheresis clinic a day before her scheduled RCE to draw a blood sample for antibody screening and crossmatching of RBC units. This 1-day lead time would often be insufficient. That is, 8–10 suitable units would not be ready the next day because the antibody testing would take at least several hours to a day, and more importantly, the order for several units of RBCs that lacked all 6 of the above antigens could not be routinely fulfilled in just one day.

The patient would show up the next day for her scheduled RCE appointment, and the apheresis nurse would discover that not all of the expected RBC units were available. Thus, the RCE would have to be delayed one or more days. The patient would be told this only after she had arrived for her RCE appointment. In sum, these last-minute cancellations caused inconveniences and treatment delays.

As different people are on apheresis service at different times and, therefore, encountered this particular patient at different times, the apheresis physicians, nurses, and blood bank technologists did not immediately notice these repeated last-minute cancellations for this patient. We credit the hematologist for bringing this problem to our attention.

While we have indicated the main problem and introduced the basics of the process, we will now describe the old process in more detail as well as the changes we made.

## 3. The Process Challenge and Improvement

### 3.1. Transfusion of SCD Patients at the UWHC

At the University of Wisconsin Hospital and Clinics (UWHC), approximately 15–20 SCD patients regularly receive outpatient RCEs at an interval of 1 to 4 months between RCEs. Currently, the UWHC blood bank (UWBB) receives its blood products from the American Red Cross (ARC). The blood center does not have access to the UWHC patient information through our electronic medical record. Finally, at the UWHC, we prefer (but do not absolutely require) RBCs that are relatively fresh (<14 days old) for SCD patients to maximize the RBC lifespan in the patient.

### 3.2. How ARC Maintains Its Inventory

The blood center has implemented several practical guidelines to maintain its inventory of blood products for SCD patients. Every day, based on the demographic information of the donors for that day, ARC identifies units that have a high probability of being used in the future for SCD patients. These include RBC phenotypes that are more common or relatively unique to the black population. These “sickle cell patient units” are stored on separate shelves for ease of access and improved organization.

Unused units are held for a maximum allowable shelf life of 42 days at the ARC. As stated earlier, relatively fresh units are preferred for RCEs for SCD patients at the UWHC. Therefore, once a unit goes past this date, it is usually not used at the UWHC but sent to other ARC branches that may need it.

### 3.3. Introduction to the Problem

After learning about the repeated delays for this patient with multiple alloantibodies, we investigated the root cause behind these delays and devised a new method to order blood products. We report both the old and new processes.

### 3.4. Old Process

The process of arranging an outpatient RCE for an SCD patient begins when a physician from the patient's clinical team (usually a hematologist) consults transfusion medicine. The transfusion medicine physician places the RCE order and communicates this to the apheresis nurse. The apheresis nurse makes an appointment for the patient.

The key step we wish to highlight is that the weekday before the RCE, the nurse instructs the UWBB to obtain the required number of compatible units. The UWBB technologist then orders the RBC units from the ARC. An antibody screen is obtained the day of the procedure or the day before, but historical data are used to informally begin the search for units. Thus, in our old process, we typically gave our blood supplier around one day advance notice. This worked well for most SCD patients who had no or few alloantibodies.

As stated earlier, the ARC does not have direct access to UWHC patient information, and therefore, the computerized transfusion appointment made would not be “visible” to the ARC. Therefore, transfusion appointments and the required number of antibody-compatible units had to be communicated separately to the ARC.

The ARC obtains the required number of antibody-compatible units from various locations. First, the ARC identifies relatively fresh compatible units on their “sickle cell shelf.” If adequate units are not available from there, our local ARC contacts other ARC branches to obtain additional units.

There were a few disadvantages and inconveniences caused by this method. The main one was that only one day was available to the ARC to identify compatible units. Moreover, due to time limitations, tradeoffs had to be made regarding the freshness of the unit. In some cases, older units had to be used. This is not optimal for the patient because of the shorter RBC lifespan. As we noted, in some instances, RCEs needed to be postponed by a few days to provide more time for the ARC to obtain enough units.

### 3.5. New Process

Once we identified the pattern in these delays, we resolved to improve our process and decrease these delays. We applied the basic principles of the theory of constraints in the field of systems engineering [12]. We identified that the key constraint was the short time available to the ARC to find units. In other words, this was the rate-limiting step that was causing delays. Thus, our goal was to increase this lead time significantly.

It was not self-evident what the best process would be. Several ideas were generated and considered. Ideally, there would be a wholly automated or computerized solution, but this is not feasible because the blood center cannot access our appointment data directly.

Thus, we devised the following new process. the UWHC and UWBB would communicate with the ARC before the scheduled outpatient RCE appointment with the following details. The process remained the same before and after the apheresis nurse's steps. However, instead of waiting until the day before the RCE, we added a step for the nurse to e-mail the UWBB's senior technologists at the same time that the nurse makes the patient's next RCE appointment. The UWBB senior tech receives this e-mail and uses this as the signal to order RBCs from the blood center about 6–8 weeks before this patient's next RCE. Thus, the order is received by the blood center at least 7 days in advance of the RCE and, in many cases, several weeks in advance.

The processes for the ARC to communicate to the UWBB and for the UWBB to communicate with the clinical teams are “push” processes. If the units are on track to be available, then no signals are sent. However, if a delay is anticipated to affect the availability of blood and the performance of the RCE at the planned time and date, then the ARC pushes this signal via e-mail to UWBB, and the UWBB in turn communicates this via the transfusion medicine resident to the clinical team.

## 4. Follow-Up

We can report anecdotally of several advantages of this new process. Since implementing this new process, the most important improvement has been that the metric of “delays due to blood procurement for outpatient RCEs” has dropped to zero. In other words, the anecdotally observed number of delays greater than 2 hours went from nonzero in the era before this intervention to zero after this intervention was implemented around 5 years before we write this.

## 5. Discussion

To our knowledge, a process to ensure the ordering of RBCs at least 7 advance of a scheduled outpatient RCE has not been reported in the medical literature. However, we do not claim that this process is necessarily novel or unique, as colleagues at other institutions may have created similar processes without publishing them.

This may seem like a relatively mundane and trivial change, but sometimes, small modifications can be leveraged to produce large effects. Namely, the lead time for the blood supplier was increased from 1 day to 7 days, and the probability of a treatment delay or last-minute cancellation by us essentially drops to zero. All this is due to a simple e-mail from the apheresis nurse to the blood bank senior technologists. The e-mails between nurses, techs, and blood supplier contacts are HIPAA compliant. Verification emails are sent to confirm receipt and next steps. Moreover, both before and after this process change, the electronic ordering process between the UWBB and the ARC is and remains a manual process.

While this process does not replace the necessary antibody screen that is usually drawn the day before the red cell exchange, the historical phenotyping and antibody specificity results allow the blood center to begin the preliminary search for suitable RBC units. In most cases, the patient's historical antibody data turn out to be the same as the data from the actual sample that is used for the official search.

One tradeoff is that this increased preparedness comes at the expense of an increased risk of wasted effort and, in the worst case of a patient cancellation or no show, potentially wasted RBC units. In other words, because we are finding units farther in advance, we rely more heavily on patient compliance in order to actually use those units for the exchange. In short, increasing the lead time inevitably decreases the window of a “penalty-free” patient cancellation. One practical implication is that this process may not be optimal for a patient who has a proven track record of no shows.

Finally, this process improvement is not directly relevant to emergency or unplanned acute RCEs. An advance notice of weeks or even hours is not feasible for such procedures. We recommend an excellent process analysis for emergency RCEs [10]. However, our key strategy of ordering RBCs as soon as possible to minimize delays from this rate-limiting step still applies to the acute setting. For example, one could create a process that prioritizes the ordering of RBCs as one of the very first steps for an acute RCE. Our literature search did not yield any articles that describe a process similar to our new process specifically for scheduled outpatient RCEs, so we share it here.

## 6. Conclusions

Despite many variations in operational practices across the world, we believe that our basic idea, maximize the lead time to the blood supplier, may be useful to any service that provides scheduled outpatient red cell exchanges.

## Data Availability

No data were used to support the findings of this study.

## Abbreviations

SCD: Sickle cell disease
RBC: Red blood cell
RCE: Red cell exchange
DHTR: Delayed hemolytic transfusion reaction
HIPAA: Health Insurance Portability and Accountability Act
HHS: Hyperhemolysis syndrome
UWHC: Wisconsin Hospital and Clinics
UWBB: UWHC blood bank
ARC: American Red Cross.
